# Factors Associated With Burnout, Post-traumatic Stress and Anxio-Depressive Symptoms in Healthcare Workers 3 Months Into the COVID-19 Pandemic: An Observational Study

**DOI:** 10.3389/fpsyt.2021.668278

**Published:** 2021-07-08

**Authors:** Samuel Cyr, Marie-Joelle Marcil, Marie-France Marin, Jean-Claude Tardif, Stéphane Guay, Marie-Claude Guertin, Camille Rosa, Christine Genest, Jacques Forest, Patrick Lavoie, Mélanie Labrosse, Alain Vadeboncoeur, Shaun Selcer, Simon Ducharme, Judith Brouillette

**Affiliations:** ^1^Research Center, Montreal Heart Institute, Montreal, QC, Canada; ^2^Faculty of Pharmacy, Université de Montréal, Montreal, QC, Canada; ^3^Department of Psychiatry and Addiction, Université de Montréal, Montreal, QC, Canada; ^4^Department of Psychology, Université du Québec à Montréal, Montreal, QC, Canada; ^5^Research Center, Institut universitaire en santé mentale de Montréal, Montreal, QC, Canada; ^6^Faculty of Medicine, Université de Montréal, Montreal, QC, Canada; ^7^Centre D'étude sur le Trauma, Centre de Recherche de l'Institut Universitaire en Santé Mentale de Montréal, Montreal, QC, Canada; ^8^Montreal Health Innovations Coordinating Center, Montreal, QC, Canada; ^9^Faculty of Nursing, Université de Montréal, Montreal, QC, Canada; ^10^Department of Organization and Human Resources, ESG UQAM, Montreal, QC, Canada; ^11^Division of Emergency Medicine, Department of Pediatrics, Centre hospitalier universitaire Sainte-Justine, Montreal, QC, Canada; ^12^Department of Psychiatry, Douglas Mental Health University Institute, McGill University, Montreal, QC, Canada; ^13^McConnell Brain Imaging Centre, Montreal Neurological Institute, McGill University, Montreal, QC, Canada

**Keywords:** burnout, anxiety, depression, post-traumatic stress disorders, COVID-19, health personnel, psychological stress

## Abstract

**Objective:** This study examined how best to identify modifiable protective and risk factors for burnout in healthcare workers in the face of the COVID-19 pandemic. Individual, occupational, organizational and social factors were investigated. The study also assessed the impact of these factors on post-traumatic stress disorder (PTSD), anxiety, and depression.

**Methods:** Healthcare workers in the Quebec (Canada) healthcare system were recruited between May 21 to June 5, 2020. Participants answered an electronic survey 3 months after the COVID-19 epidemic outbreak began in Canada. Using the Maslach Burnout Inventory, PTSD Checklist for DSM-5, and Hospital Anxiety and Depression Scale, we studied the prevalence of burnout, PTSD, anxiety and depression in this cohort. Multivariable logistic or linear regression models including resilience, social and organizational support, workload and access to mental health help, simulation techniques and protective personal equipment (PPE) as well as perception of PPE security were conducted for each outcome.

**Results:** In mid-June 2020, 467 participants completed the survey. We found that half (51.8%) of the respondents experienced burnout characterized by emotional exhaustion and/or depersonalization at least once a week. In total, 158 healthcare workers (35.6%) displayed severe symptoms of at least one of the mental health disorders (24.3% PTSD, 23.3% anxiety, 10.6% depression). Resilience (OR = 0.69, 95% CI: [0.55–0.87]; *p* = 0.002) and perceived organizational support (OR = 0.75, 95% CI: [0.61–0.93]; *p* = 0.009) were significantly associated with burnout and other outcomes. Social support satisfaction, perception of PPE security, work type and environment, mental health antecedents and reassignment were associated with PTSD and/or anxiety and/or depression, but not burnout.

**Conclusion:** Future studies should address primarily resilience and perceived organizational support to promote mental health and prevent burnout, PTSD, anxiety and depression.

## Introduction

As of May 6, 2021, over 155 million people have been affected by the SARS-CoV2 virus and over 3 million people have died after developing COVID-19 (https://coronavirus.jhu.edu/map.html). Healthcare workers are at high risk of contracting SARS-CoV2 (1) and constitute around 10% of all confirmed cases in North America, roughly 10 to 20% in European countries and nearly 5% in China (2). In the province of Quebec, Canada, 17.2% of COVID-19 confirmed cases were medical workers (3). In addition to managing their own health and that of the population, they have to cope with rapidly changing organizational, occupational, and familial functioning. This has placed additional pressure on these professionals, in whom 30% reported burnout (4, 5) prior to the COVID-19 pandemic. Burnout is not officially considered a mental health disorder (unlike depression) but is rather defined as an occupational phenomenon “resulting from chronic workplace stress that has not been successfully managed” (6). It is characterized by “feelings of energy exhaustion; increased mental distance from work; and reduced professional efficacy.” Knowledge of the psychological impacts of the COVID-19 pandemic on health professionals, mostly physicians and nurses, is rapidly emerging (7, 8). A few studies have directly addressed burnout symptoms (9–25), including a recent meta-analysis that reported a pooled prevalence of 37.4% among healthcare workers (26).

Pre-pandemic research on burnout in healthcare workers has identified neuroticism, high workload, value incongruence, and poor job climate as risk factors (22, 27–30). In contrast, higher perceived social support and job resources protect against it (22, 27–30). Considering the impact of burnout on both the professional and personal lives of healthcare workers as well as the potential negative impact on the quality of care provided to patients (31), it is important to study the factors associated with burnout following the COVID-19 pandemic. To our knowledge, no study combined in the same analysis organizational [e.g., perceived organizational support, defined by the extent in which the organization values contributions and cares about well-being (32)], occupational (e.g., workload) and individuals [e.g., resilience, defined by positive adjustment in response to stress or trauma and measured with Connor-Davidson Resilience Scale (33)] as potential factors. Moreover, social support has been infrequently studied in the recent COVID-19 literature on burnout among healthcare workers, with only one study reporting it as a factor to be considered (34). The primary objective of our study is to identify modifiable protective and risk factors associated with burnout in a wide range of healthcare workers facing the COVID-19 pandemic. Individual, occupational, organizational, and social factors will be investigated with the objective to determine the most promising field to address for future interventions. Secondarily, we aim to study the impact of the same factors on post-traumatic stress disorder (PTSD), anxiety, and depression. Knowledge of potential protection and risk factors is crucial to roll out strategies to limit the impact of other crises similar to the COVID-19 pandemic.

## Methods

### Design

This cross-sectional study was approved by the ethics committees of the Montreal Heart Institute on May 14th, 2020. It comprises an electronic survey performed at 3 months (June 2020) after the start of the COVID-19 epidemic outbreak in Quebec, Canada (March 2020).

The Montreal Health Innovations Coordination Center (MHICC) specifically developed the web platform for the present study. The system used the MHICC extranet portal with secure access to web pages by a Transport Layer Security (TLS) certificate, using standard encryption technology (2,048 bit private key length and 128-bit bulk encryption key length) and an auto logoff function in the event of a sustained period of inactivity. The data collected using this platform was sent and saved to the MHICC for a period of 10 years. Each participant only had access to their own data, and in no case had access to the data of other participants. The platform was accessible in French and in English via a smartphone, a tablet or a computer.

Between May 21st and June 5th 2020, the study team recruited healthcare workers from across Quebec's healthcare system, including those in long-term care centers (in French: Centres d'hébergement de soins de longue durée [CHSLD]). To do so, we shared a newsletter explaining the objectives of the study through study collaborators' social media accounts, Quebec health professional organizations and association's networks, and conventional media. The newsletter directed the interested healthcare workers to a web page that served as an initial screening for eligibility for participation. All participants were required to have access to an e-mail address and the Internet, had to be age 18 or over and had to work as one of the listed health care work types (administrative agent, beneficiary attendant, doctor/resident doctor, laboratory technician, kitchen attendant, maintenance agent, administrator, nurse, other health professional [occupational therapists, respiratory therapists, nutritionists, psychologists, social workers, etc.]). After reading the information and consent form, the users could sign it, if desired, to become participants in the study. In addition to the survey, we asked participants if they wanted to be contacted by the research team for (1) a post-survey follow-up to collect their comments and/or questions and/or (2) feedback on their individual results if the questionnaires indicated risks of developing PTSD, anxiety or depression (while facilitating access to psychology or other mental health resources). In addition, all participants could contact the research team by phone or e-mail if they had any questions related to the research project or if they needed either psychology referral, or referral to any other mental health organizations. It should be noted that we did not use the snowball sampling technique here since the subjects who consented to participate were not invited to continue recruiting among their affiliation (35, 36).

In mid-June 2020, study coordinators sent one to three e-mails to invite participants to complete the 3-month online survey. Participants had 1 week to respond and partially completed surveys were accepted.

### Measures

The main features of the tools used are presented here, but additional details about the surveyed measures are described in Supplementary Table A1 of Appendix 1. The presence of burnout was studied using the Maslach Burnout Inventory (MBI 2). The questions evaluate two dimensions of burnout syndrome, namely emotional exhaustion (“I feel burned out from my work”) and depersonalization (“I have become more callous toward people”) on a scale ranging from 0 (“never”) to 6 (“everyday”). The participant must experience at least weekly emotional exhaustion and/or depersonalization to be considered burned out (37, 38). This questionnaire has an adequate reliability, with an internal consistency of α = 0.80 (39). PTSD, anxiety, and depression symptoms were studied as continuous variables. PTSD was checked using total scores on the PTSD Checklist for DSM-5 (PCL-5), a 20-item self-report measure that assesses symptoms of PTSD with a high internal consistency (α = 0.94) (40, 41). PCL-5 corresponds mainly to the DSM-5 criteria with a variation of criterion A. Indeed, “exposure to actual death or threatened death, serious injury, or sexual violence” (42) is replaced by a “very stressful experience” without specification about COVID-19 context. Each item is rated on a scale of 0 to 4 and a score is generated (0–80). Anxiety and depression symptoms were verified using the Hospital Anxiety and Depression Scale (HADS), a questionnaire composed of 14 items in total, separated into two scales of 7 items each (anxiety and depression). Internal consistency is high for both HADS-A (α = 0.83) and for HADS-D (α = 0.82) (43–45). Items are graded on a scale of 0 to 3 and a score is produced for each of the two sub-scales (0–21). We used cut-off scores of 31 or more for PCL-5, and 11 or more for each component of HADS, as clinically significant PTSD (40, 46, 47), anxiety or depression symptoms (45).

Resilience, self-compassion, social and organizational support were measured with the Connor-Davidson Resilience Scale [α = 0.85 (33)], the Self-Compassion Scale [α = 0.91 (48)], the Social Support Questionnaire [α = 0.79 (49)] and the Perceived Organizational Support Questionnaire [α = 0.93 (50)], respectively. We also measured access to personal protective equipment (PPE) and feeling of security “When you used personal protective equipment as part of your duties, you felt [Totally safe to Totally at risk].” Socio-demographic (including sex), work type (including intensive care or emergency work, direct care of COVID patients, and reassignment), type of work environment, workload, medical characteristics (including COVID status), access to simulation technique and mental help data were also collected.

### Sample Size Calculation

We calculated the sample size based on a simple logistic regression model with resilience as the single independent variable of interest. Then, we made a correction based on the correlation between the variable of interest and the model's other independent variables. The expected overall burnout rate at 3 months was 50% (21). Using a simple logistic regression model with one continuous independent variable of interest at a two-sided 0.05 significant level, a sample size of 285 participants would provide 80% power to detect an odds ratio of 0.72 for an increase of one standard deviation (SD) of the independent variable. This odds ratio is comparable to the one reported in a study on burnout and work-life balance satisfaction of physicians and the general US working population (5). In the context that our recruitment was successful, we then decided to increase our sample size objective to 500 participants. Using the same assumption as stated before (50% burnout rate), a sample size of 500 participants would provide 80% power to detect an odds ratio of 0.75 for an increase of one SD of the independent variable with a simple logistic regression at a two-sided 0.05 significant level.

### Analyses

Study characteristics were summarized using counts and percentages for categorical variables and mean ± SD for continuous variables. To identify the survey response rate, we divided the number of participants who completed the survey by the total number of participants who consented to participate in the study and then multiplied by 100 to obtain a percentage. For each efficacy endpoint (burnout, PTSD, anxiety or depression), a multivariable logistic (or linear) regression model including all the pre-specified independent variables of clinical interest (resilience, social support, workload, perceived organizational support, access to mental health help, access to PPE, feeling of security using PPE and access to simulation techniques) was fit first. No variable selection was done at this stage and all independent variables were included. The pre-specified adjustment variables (psychiatric antecedents, type of employment, intensive care or emergency work, type of environment, COVID status, direct care of COVID patients, reassignment, sex) showing a *p*-value lower than 0.2 in univariate models were then identified and entered in the previous model including all independent variables (which were forced in the model) using the stepwise procedure. The criteria for an adjustment variable to stay in the final multivariable model was a significance level of 0.05. For each endpoint, an exploratory analysis was conducted by adding the self-compassion variable to the final multivariable model identified above. Adjusted odds ratio for logistic regressions and adjusted coefficients for linear regressions were calculated with 95% confidence intervals.

Scores for each questionnaire were calculated according to the formulas provided in the Supplementary Material. For incomplete questionnaires, it was still possible to calculate scores provided that no more than a pre-specified number of individual questions were unanswered. Otherwise, the scores were considered missing. No imputation was done for missing data. Before using multivariable regression models, the variables were closely examined for outliers, distribution issues or sparse data, and issues were fixed prior to running any statistical analysis. We also verified correlations between the pre-specified independent variables and looked at variance inflation factors to identify possible multicollinearity problems, but none were found. In addition, basics assumptions of the proposed analyses, such as linearity, were checked and all assumptions were met. All statistical tests were two sided and conducted at a 0.05 significance level. Analyses were performed with the use of SAS release 9.4 [SAS Institute Inc., Cary, NC, USA].

## Results

Of the 564 participants, 467 (83%) respondents completed the 3-month survey. Two (0.4%) participants have withdrawn, one before and one after completing the 3-month survey.

Table 1 shows socio-demographic and occupational characteristics of the 467 participants, as well as COVID-19 personal and occupational demographics. The vast majority were Caucasian (93.5%) and were female sex (89.4%). More than half worked between 35 and 44 h per week, and almost all of them were still employed in the healthcare system as of June 2020. Regarding work types, nurses, physicians, and other health professionals (e.g., respiratory or occupational therapists, psychologists, social workers) accounted for three quarters of the sample; administrators, administrative agents, laboratory technicians, beneficiary attendants, and paramedics constituted the other quarter. Participants worked in diverse work environments including, in descending order, hospitals, local community service centers (in French: *Centres locaux de services communautaires* [CLSC]), clinics and CHSLD. About a quarter of participants worked in another environment such as rehabilitation centers or child and youth protection centers. Ninety-one percent of the respondents perceived they had access to psychological resources if needed. Concerning the PPE, 67% of the respondents always had access to it, with a majority feeling either “pretty” or “totally safe” using it. Twenty-two percent of respondents participated in COVID-related simulation-type practices, the last simulation round having occurred 1–2 months ago for 50% of these participants. Seventy percent of the respondents had been tested for SARS-CoV2 and <5% of them had received a positive status. Close to 40% of respondents were involved in direct COVID care and 33% were reassigned either to another practice area or establishment (51).

**Table 1:** Socio-demographic, occupational and COVID-19 specific characteristics of participants.

**Variables**	**Mean ± SD or *n* (%)**	**All *n* = 467**
**Age** (years)	39 ± 9	461
**Female sex**	413 (89.4%)	462
**Ethnicity**		463
Caucasian	433 (93.5%)	
Hispanic	3 (0.6%)	
Black	4 (0.9%)	
Asian	9 (1.9%)	
Native American	2 (0.4%)	
Two of the above	12 (2.6%)	
**Marital status**		464
Never married	78 (16.8%)	
Married/Re-married	136 (29.3%)	
Separated/Divorced	28 (6.0%)	
Common-law	201 (43.3%)	
Widowed	1 (0.2%)	
Other	20 (4.3%)	
**Parental status**		464
Yes	290 (62.5%)	
**Antecedent of psychiatric disorder**		467
Yes	128 (27.4%)	
**Work type**		464
Administrator	17 (3.7%)	
Administrative agent	16 (3.4%)	
Beneficiary attendant	11 (2.4%)	
Laboratory technician/technologist	8 (1.7%)	
Nurse	117 (25.2%)	
Other health professional (ergotherapist, respiratory therapist, psychologist, social worker, etc.)	140 (30.2%)	
Paramedics	11 (2.4%)	
Physician	104 (22.4%)	
Resident physician	10 (2.2%)	
Other	30 (6.5%)	
**Work environment**		464
CLSC	57 (12.3%)	
CHSLD	26 (5.6%)	
University Health Center	139 (30.0%)	
Non-University Health Center	78 (16.8%)	
Medical clinic	38 (8.2%)	
Other	126 (27.2%)	
**Intensive care or emergency work**		461
Yes	69 (15.0%)	
**Workload**		455
34 h or less	80 (17.6%)	
35–44 h	265 (58.2%)	
45–54 h	67 (14.7%)	
55–64 h	28 (6.2%)	
65 h or more	15 (3.3%)	
**Current work status** (as of June 2020)		464
Still a worker in the Quebec health system	455 (98.1%)	
Employee of another employer	1 (0.2%)	
Student	2 (0.4%)	
Retired	1 (0.2%)	
Absent from the labor market	1 (0.2%)	
Other	4 (0.9%)	
**Access to mental help**		461
Yes	420 (91.1%)	
**Types of mental help professional**		420
Psychologist	97 (23.0%)	
Psychotherapist	12 (2.9%)	
Social worker	9 (2.1%)	
Family doctor	55 (13.1%)	
Employee assistance program	222 (52.7%)	
Other	26 (6.2%)	
**Access to PPE**		454
Never or rarely	15 (3.3%)	
Sometimes	26 (5.7%)	
Often	109 (24.0%)	
Always	304 (67.0%)	
**Perception of security using PPE**		451
Totally safe	66 (14.6%)	
Pretty safe	331 (73.4%)	
Rather or totally in danger	54 (12.0%)	
**Participation in any simulation-type practices**		462
Yes	100 (21.6%)	
**Last simulation round**		100
<1 week	2 (2.0%)	
<1 month	17 (17.0%)	
1–2 months ago	50 (50.0%)	
<6 months	31 (31.0%)	
**COVID status**		461
Negative	302 (65.5%)	
Recovered	19 (4.1%)	
Never been tested	140 (30.4%)	
**Direct COVID patient care**		462
Yes	182 (39.4%)	
**Reassignment**		462
Yes	151 (32.7%)	

*CHSLD, Long-term care center; CLSC, Local community service center; PPE, personal protective equipment*.

The scores for the psychological questionnaires included in the 3-month survey are presented in Table 2. Half of the respondents (51.8%) experienced at least weekly emotional exhaustion and/or depersonalization on the Maslach Burnout Inventory. In total, 158 different individuals (35.6%) displayed severe symptoms of at least one of the mental health disorders; 24.3% of respondents displayed severe symptoms of PTSD, 23.3% of anxiety, and 10.6% of depression. Mean scores of resilience, social support satisfaction, perceived organizational support, and self-compassion are also presented in Table 2.

**Table 2:** Psychological questionnaire scores of participants.

		**All *n* = 467**
**Burnout** (MBI), yes	236 (51.8%)	456
**Post-traumatic stress symptoms** (PCL-5)	19.8 ± 15.0	453
≥31	110 (24.3%)	
**Anxiety symptoms** (HADS-A)	7.9 ± 4.0	447
≥11	104 (23.3%)	
**Depressive symptoms** (HADS-D)	5.2 ± 3.9	445
≥11	47 (10.6%)	
**Severe mental health symptoms**, yes	158 (35.6%)	444
**Resilience** (CD-RISC)	27.4 ± 6.2	456
**Social support questionnaire**		
Satisfaction	28.7 ± 6.1	455
**Perceived organizational support scale**	22.5 ± 11.6	456
**Self-compassion scale**	12.1 ± 3.7	455

*Scores are presented as Mean ± SD or n (%). Severe mental health symptoms are defined as post-traumatic stress (PCL-5 ≥ 31), anxiety (HADS-A ≥11) or depressive (HADS-D ≥11) symptoms. CD-RISC, Connor-Davidson Resilience Scale; HADS, Hospital Anxiety and Depression Scale; MBI, Maslach Burnout Inventory; PCL-5, Post-traumatic Stress Disorder Checklist for DSM-5*.

Table 3 presents the results of the final multivariable logistic regression model for burnout status. Resilience (OR = 0.69, 95% CI: [0.55–0.87]; *p* = 0.002) and perceived organizational support (OR = 0.75, 95% CI: [0.61–0.93]; *p* = 0.009) were the only two variables significantly associated with burnout, in an inverse relationship. In other words, there is a 31% decrease in the odds of burnout for each SD (6) increase on the resilience scale; and there is a 25% decrease in the odds of burnout for each SD (12) increase on the perceived organizational support scale.

**Table 3:** Adjusted odds ratios, 95% confidence interval and *p*-values from multivariable logistic regression analysis for burnout among healthcare workers (*n* = 424).

	**Variables**	**OR**	**95% CI**		***p***
**Independent**	**Resilience**	0.69	0.55	0.87	0.002
	**Social support**	0.85	0.69	1.06	0.15
	**Workload**				0.78
	[35–44] h vs. ≤ 34 h	0.82	0.47	1.41	0.47
	[45–54] h vs. ≤ 34 h	0.88	0.43	1.79	0.72
	[55–64] h vs. ≤ 34 h	0.73	0.27	1.97	0.54
	≥ 65 h vs. ≤ 34 h	1.58	0.46	5.50	0.47
	**Perceived organizational support**	0.75	0.61	0.93	0.009
	**Access to simulation technique** (yes vs. no)	1.05	0.64	1.74	0.84
	**Access to mental health help** (yes vs. no)	0.84	0.40	1.75	0.64
	**Access to PPE**				0.23
	Sometimes vs. never or rarely	2.62	0.41	16.90	0.31
	Often vs. never or rarely	4.58	0.86	24.47	0.08
	Always vs. never or rarely	3.40	0.66	17.60	0.14
	**PPE perception of security**				0.40
	Pretty safe vs. totally safe	0.81	0.45	1.45	0.47
	Rather in danger or totally at risk vs. totally safe	1.27	0.52	3.08	0.60

*ORs are presented for an increase of one standard deviation (SD) for continuous variables (resilience; SD = 6.06, social support; SD = 5.93, and perceived organizational support; SD = 11.68). CI, Confidence intervals; OR, Odds ratio; PPE, Personal protective equipment*.

Multivariable linear regression analyses were performed for PTSD, anxiety, and depression symptoms (Tables 4–6). Table 4 shows that resilience, social support satisfaction, perception of organizational support and perception of security using PPE were inversely associated with the severity of PTSD symptoms. In regard to employment types, administrative agents, administrators, other health professionals, laboratory technicians, beneficiary attendants and nurses displayed on average higher PTSD estimate scores compared to physicians. Presence of previous psychiatric conditions was positively associated with PTSD symptoms. The Table 5 shows that resilience, social support satisfaction and perceived organizational support were inversely associated with the severity of anxiety symptoms. Regarding work environments, CHSLD displayed on average significantly higher scores on the anxiety scale compared to university health centers. Non-university health center workers displayed on average significantly lower scores on the anxiety scale compared to university health centers. None of the other work environments were different in terms of anxiety compared to university health centers. Previous psychiatric conditions was significantly and positively associated with the severity of anxiety symptoms. Table 6 shows that resilience, social support, perceived organizational support, work type, reassignment, and psychiatric antecedents were significantly associated with depression symptoms' severity. Table 7 highlights significant findings across all efficacy endpoints.

**Table 4:** Adjusted coefficient, 95% confidence interval and *p*-values from multivariable linear regression analysis for PTSD symptoms among healthcare workers (*n* = 426).

	**Variables**	**Coefficient**	**95% CI**		***p***
**Independent**	**Resilience**	−1.91	−3.19	−0.64	0.003
	**Social support**	−3.21	−4.41	−2.00	<0.0001
	**Workload**				0.31
	[35–44] h vs. ≤ 34 h	−0.90	−3.99	2.20	0.57
	[45–54] h vs. ≤ 34 h	1.06	−3.11	5.22	0.62
	[55–64] h vs. ≤ 34 h	4.76	−0.96	10.48	0.10
	≥ 65 h vs. ≤ 34 h	0.70	−6.35	7.75	0.85
	**Perceived organizational support**	−2.53	−3.81	−1.25	0.0001
	**Access to mental health help** (yes vs. no)	−0.15	−4.25	3.95	0.94
	**Access to PPE**				0.25
	Sometimes vs. never or rarely	−7.75	−17.07	1.58	0.10
	Often vs. never or rarely	−4.50	−12.48	3.47	0.27
	Always vs. never or rarely	−6.17	−13.99	1.66	0.12
	**PPE perception of security**				<0.0001
	Pretty safe vs. totally safe	−0.51	−3.89	2.88	0.77
	Rather in danger or totally at risk vs. totally safe	8.38	3.47	13.28	0.0009
	**Access to simulation technique** (yes vs. no)	1.82	−1.09	4.73	0.22
**Adjustment**	**Work type**				0.0004
	Administrative agent vs. physician	14.32	6.90	21.74	0.0002
	Other vs. physician	2.64	−2.51	7.78	0.31
	Administrator vs. physician	9.93	3.55	16.30	0.002
	Nurse vs. physician	6.11	2.61	9.60	0.0006
	Resident physician vs. physician	1.37	−6.90	9.64	0.75
	Paramedics vs. physician	6.72	−0.96	14.40	0.09
	Other health professional vs. physician	3.58	0.05	7.11	0.047
	Beneficiary attendant vs. physician	11.29	3.36	19.22	0.01
	Laboratory technician/technologist vs. physician	8.84	0.10	17.58	0.048
	**Psychiatric antecedent** (yes vs. no)	9.75	7.02	12.48	<0.0001

*Regression coefficients are presented for an increase of one standard deviation (SD) for continuous variables (resilience; SD = 6.20, social support; SD = 6.09, and perceived organizational support; SD = 11.60). CI, Confidence intervals; PPE, Personal protective equipment; PTSD, Post-traumatic stress disorder*.

**Table 5:** Adjusted coefficient, 95% confidence interval and *p*-values from multivariable linear regression analysis for anxiety scores among healthcare workers (*n* = 421).

	**Variables**	**Coefficient**	**95% CI**		***p***
**Independent**	**Resilience**	−1.02	−1.37	−0.66	<0.0001
	**Social support**	−0.49	−0.82	−0.15	0.004
	**Workload**				0.38
	[35–44] h vs. ≤ 34 h	−0.57	−1.44	0.31	0.20
	[45–54] h vs. ≤ 34 h	−0.08	−1.21	1.04	0.88
	[55–64] h vs. ≤ 34 h	0.60	−0.94	2.15	0.44
	≥65 h vs. ≤ 34 h	0.06	−1.87	1.99	0.95
	**Perceived organizational support**	−0.67	−1.02	−0.31	0.0003
	**Access to mental health help** (yes vs. no)	−0.28	−1.44	0.88	0.63
	**Access to PPE**				0.42
	Sometimes vs. never or rarely	−1.75	−4.44	0.95	0.20
	Often vs. never or rarely	−1.17	−3.48	1.15	0.32
	Always vs. never or rarely	−1.56	−3.83	0.70	0.18
	**PPE perception of security**				0.06
	Pretty safe vs. totally safe	−0.33	−1.27	0.62	0.50
	Rather in danger or totally at risk vs. totally safe	0.99	−0.40	2.38	0.16
	**Access to simulation technique** (yes vs. no)	−0.17	−0.98	0.65	0.69
**Adjustment**	**Work environment**				0.02
	Other vs. University Health Center	−0.03	−0.91	0.84	0.94
	CHSLD vs. University Health Center	1.53	0.03	3.04	0.046
	CLSC vs. University Health Center	0.54	−0.63	1.71	0.36
	Non-University Health Center vs. University Health Center	−1.02	−2.00	−0.04	0.04
	Medical clinic vs. University Health Center	−0.59	−1.82	0.64	0.34
	**Psychiatric antecedent** (yes vs. no)	2.36	1.59	3.12	<0.0001

*Regression coefficients are presented for an increase of one standard deviation (SD) for continuous variables (resilience; SD = 6.20, social support; SD = 6.09, and perceived organizational support; SD = 11.60). CHSLD, Long-term care center; CI, Confidence intervals; CLSC, Local community service center; PPE, Personal protective equipment*.

**Table 6:** Adjusted coefficient, 95% confidence interval and *p*-values from multivariable linear regression analysis for depression scores among participants (*n* = 419).

	**Variables**	**Coefficient**	**95% CI**		***p***
**Independent**	**Resilience**	−1.00	−1.32	−0.68	<0.0001
	**Social support**	−0.95	−1.25	−0.65	<0.0001
	**Workload**				0.052
	[35–44] h vs. ≤ 34 h	0.32	−0.46	1.10	0.42
	[45–54] h vs. ≤ 34 h	0.99	−0.06	2.04	0.06
	[55–64] h vs. ≤ 34 h	1.91	0.48	3.34	0.01
	≥65 h vs. ≤ 34 h	1.57	−0.19	3.32	0.08
	**Perceived organizational support**	−0.59	−0.91	−0.27	0.0003
	**Access to mental health help** (yes vs. no)	0.22	−0.81	1.25	0.67
	**Access to PPE**				0.56
	Sometimes vs. never or rarely	−1.48	−3.90	0.94	0.23
	Often vs. never or rarely	−0.80	−2.88	1.28	0.45
	Always vs. never or rarely	−1.07	−3.11	0.96	0.30
	**PPE perception of security**				0.16
	Pretty safe vs. totally safe	0.08	−0.76	0.93	0.84
	Rather in danger or totally at risk vs. totally safe	1.01	−0.21	2.23	0.10
	**Access to simulation technique** (yes vs. no)	0.25	−0.48	0.99	0.50
**Adjustment**	**Work type**				0.02
	Administrative agent vs. physician	2.51	0.66	4.36	0.01
	Other vs. physician	0.21	−1.07	1.49	0.75
	Administrator vs. physician	1.59	−0.04	3.22	0.06
	Nurse vs. physician	1.31	0.43	2.19	0.004
	Resident physician vs. physician	−0.37	−2.42	1.69	0.73
	Paramedics vs. physician	0.99	−0.92	2.91	0.31
	Other health professional vs. physician	0.59	−0.29	1.48	0.19
	Beneficiary attendant vs. physician	2.59	0.62	4.56	0.01
	Laboratory technician/technologist vs. physician	1.25	−0.92	3.43	0.26
	**Reassignment (yes vs. no)**	0.71	0.09	1.32	0.02
	**Psychiatric antecedent (yes vs. no)**	1.75	1.07	2.44	<0.0001

*Regression coefficients are presented for an increase of one standard deviation (SD) for continuous variables (resilience; SD = 6.20, social support; SD = 6.09, and perceived organizational support; SD = 11.60). CI, Confidence intervals; PPE, Personal protective equipment*.

**Table 7:** Summary table of factors significantly associated with burnout, post-traumatic stress disorder, anxiety and depression.

		**Dependent variables**		
		**Burnout**	**PTSD**	**Anxiety**	**Depression**	
**Independent variables**	Resilience	Y	Y	Y	Y
	Social support	N	Y	Y	Y
	Workload	N	N	N	N
	Perceived organizational support	Y	Y	Y	Y
	Access to mental health help	N	N	N	N
	Access to PPE	N	N	N	N
	PPE perception of security	N	Y	N	N
	Access to simulation technique	N	N	N	N
**Adjustment variables**	Sex	–	–	–	–
	Psychiatric antecedent	–	Y	Y	Y
	Type of employment	–	Y	–	Y
	Intensive care or emergency work	–	–	–	–
	Work environment	–	–	Y	–
	Participant's COVID status	–	–	–	–
	Direct COVID care	–	–	–	–
	Reassignment	–	–	–	Y

*N, Not significant; PPE, Personal protective equipment; PTSD, post-traumatic stress disorder; Y, Yes (statistically significant); –, adjustment variable not selected in the step-wise procedure*.

For the exploratory analyses, only the PTSD and anxiety models are presented, as they are the two models that have retained the self-compassion variable (SD = 3.74) in their regression models (see Tables 8, 9). There was a significant and negative association between self-compassion and PTSD (Est: −1.56, 95% CI: [−3.03 to −0.08], *p* = 0.04) and anxiety symptoms (Est: −0.82, 95% CI: [−1.23 to −0.41], *p* < 0.0001).

**Table 8:** Adjusted coefficient, 95% confidence interval and *p*-values from multivariable linear regression model with self-compassion variable for PTSD symptoms among healthcare workers (*n* = 425).

	**Variables**	**Coefficient**	**95% CI**		**p**
**Independent**	**Resilience**	−1.27	−2.68	0.14	0.08
	**Social support**	−3.01	−4.22	−1.79	<0.0001
	**Workload**				0.29
	[35–44] h vs. ≤ 34h	−1.04	−4.13	2.06	0.51
	[45–54] h vs. ≤ 34h	0.77	−3.39	4.93	0.72
	[55–64] h vs. ≤ 34h	4.73	−0.98	10.43	0.10
	≥ 65h vs. ≤ 34h	0.42	−6.61	7.45	0.91
	**Perceived organizational support**	−2.30	−3.59	−0.996	0.0006
	**Access to mental health help** (yes vs. no)	−0.17	−4.26	3.92	0.94
	**Access to PPE**				0.22
	Sometimes vs. never or rarely	−7.54	−16.85	1.77	0.11
	Often vs. never or rarely	−4.56	−12.52	3.39	0.26
	Always vs. never or rarely	−6.43	−14.25	1.38	0.11
	**PPE perception of security**				<0.0001
	Pretty safe vs. totally safe	−0.34	−3.73	3.05	0.84
	Rather in danger or totally at risk vs. totally safe	8.65	3.74	13.55	0.0006
	**Access to simulation technique** (yes vs. no)	2.01	−0.90	4.91	0.17
**Adjustment**	**Work type**				0.0003
	Administrative agent vs. physician	14.68	7.27	22.09	0.0001
	Other vs. physician	2.82	−2.37	8.02	0.29
	Administrator vs. physician	10.14	3.78	16.50	0.002
	Nurse vs. physician	6.07	2.59	9.55	0.0007
	Resident physician vs. physician	0.51	−7.78	8.79	0.90
	Paramedics vs. physician	6.89	−0.77	14.55	0.07
	Other health professional vs. physician	3.88	0.34	7.41	0.03
	Beneficiary attendant vs. physician	11.43	3.52	19.34	0.005
	Laboratory technician / technologist vs. physician	8.63	−0.09	17.35	0.052
	**Psychiatric antecedent** (yes vs. no)	9.67	6.95	12.39	<0.0001
	**Self–compassion**	−1.56	−3.03	−0.08	0.04

*Regression coefficients are presented for an increase of one standard deviation (SD) for continuous variables (resilience; SD = 6.20, social support; SD = 6.09, perceived organizational support; SD = 11.60, and self-compassion; SD = 3.74). CI, Confidence intervals; PPE, Personal protective equipment; PTSD, Post-traumatic stress disorder*.

**Table 9:** Adjusted coefficient, 95% confidence interval and *p*-values from multivariable linear regression model with self-compassion variable for anxiety symptoms among healthcare workers (*n* = 420).

	**Variables**	**Coefficient**	**95% CI**		***p***
**Independent**	**Resilience**	−0.67	−1.07	−0.28	0.0008
	**Social support**	−0.38	−0.71	−0.05	0.03
	**Workload**				0.44
	[35–44] h vs. ≤ 34 h	−0.58	−1.44	0.28	0.19
	[45–54] h vs. ≤ 34 h	−0.28	−1.38	0.83	0.63
	[55–64] h vs. ≤ 34 h	0.53	−0.98	2.05	0.49
	≥ 65 h vs. ≤ 34 h	−0.12	−2.02	1.77	0.90
	**Perceived organizational support**	−0.51	−0.87	−0.16	0.005
	**Access to mental health help** (yes vs. no)	−0.21	−1.35	0.92	0.71
	**Access to PPE**				0.38
	Sometimes vs. never or rarely	−1.38	−4.03	1.26	0.30
	Often vs. never or rarely	−1.00	−3.27	1.27	0.39
	Always vs. never or rarely	−1.49	−3.71	0.73	0.19
	**PPE perception of security**				0.03
	Pretty safe vs. totally safe	−0.35	−1.28	0.59	0.46
	Rather in danger or totally at risk vs. totally safe	1.08	−0.28	2.45	0.12
	**Access to simulation technique** (yes vs. no)	−0.12	−0.92	0.68	0.76
**Adjustment**	**Work environment**				0.01
	Other vs. University Health Center	0.09	−0.77	0.95	0.83
	CHSLD vs. University Health Center	1.49	0.02	2.97	0.048
	CLSC vs. University Health Center	0.51	−0.63	1.66	0.38
	Non–University Health Center vs. University Health Center	−1.12	−2.08	−0.16	0.02
	Medical clinic vs. University Health Center	−0.56	−1.77	0.65	0.36
	**Psychiatric antecedent** (yes vs. no)	2.31	1.56	3.06	<0.0001
	**Self–compassion**	−0.82	−1.23	−0.41	<0.0001

*Regression coefficients are presented for an increase of one standard deviation (SD) for continuous variables (resilience; SD = 6.20, social support; SD = 6.09, perceived organizational support; SD = 11.60, and self-compassion; SD = 3.74). CHSLD, Long-term care center; CI, Confidence intervals; CLSC, Local community service center; PPE, Personal protective equipment*.

## Discussion

Three months after the start of the COVID-19 pandemic, we surveyed the psychological health of 467 workers of Quebec's healthcare system. Among them, 52% met the cutoff score for burnout, much higher than pre-pandemic periods [which was estimated at ~30% (4, 5)].

Regarding psychopathologies, 24% of participants displayed clinically significant symptoms for PTSD, 23% for anxiety, and 11% for depression. These rates are consistent with those reported in the recent COVID-19 international literature on healthcare workers (7, 8), but are surprisingly similar to the rates reported before the pandemic (4). One hypothesis for this unanticipated result is the fact the thresholds to distinguish symptoms of psychopathology in self-administered questionnaires are widely heterogeneous, as are the questionnaires used (52). A final explanation is that the 3-month time point is possibly too early to detect an increase in anxio-depressive or PTSD symptoms (53). Such severe symptoms might develop months later, after initial coping mechanisms weaken.

As to factors associated with burnout and psychopathologies, in the context of COVID-related research, female sex (54), reported negative impact of work on household activities (21), urban living (55), a nursing position (56), higher exposure to COVID risks (21) and feeling pushed beyond training (21) are associated with adverse psychological outcomes. Conversely, high resilience, social support and availability of protective equipment are associated with lower levels of anxiety, burnout and insomnia (21, 56, 57). Additionally, past crises that generated important sources of strain for healthcare workers have shown the importance of verifying the impact of not only individual factors but also organizational ones on the development of burnout and psychopathologies (58–60). This proved to be the case in our study with higher resilience (individual factor) and perceived organizational support (organizational factor) being the only two variables significantly associated with better outcomes in both burnout and psychopathologies (PTSD, anxiety, depression), out of the eight independent variables verified. More particularly for burnout, they outweighed all six other independent variables as no other adjusted variables were needed to improve the model (social support, workload, access to mental help, PPE, or simulation technique, and perception of security using PPE). For PTSD, anxiety and depression symptoms, social support added to resilience and perceived organizational support in the final model as significant variables associated with symptom severity. This is consistent with results from recent COVID-19 literature; there is an inverse correlation between social support and anxio-depressive and PTSD symptoms. Surprisingly or not, social support was not significantly associated with burnout. During a notably stressful period at work, it is possible that burnout would be more strongly associated with organizational rather than social support, especially in a time of confinement.

Perception of low security while using PPE— and not lack of access *per se* —was associated with higher PTSD, but not with burnout or depression. Not feeling safe in the face of COVID-19 can lead to fear of becoming infected, potentially dying or infecting a patient or a loved one. As being exposed to threatened death is a cardinal criterion of PTSD, this can explain why these associations are specific to this fear-related conditions and do not hold for burnout, depression and anxiety symptoms (42). Depression was the only mental health outcome that was associated with reassignment. The loss of reference and network, having to learn a new working method, the resulting fatigue and feeling isolated can have contributed to this association.

In the final regression models, psychiatric antecedents were significantly associated with PTSD, anxiety and depression, but not burnout. For the latter, its effect was not strong enough as soon as it was combined with resilience and perceived organizational support. This reinforces the idea that burnout is not a mental disease. It also explicitizes that resilience and perceived organizational support can be protective in individual with and without psychiatric antecedents. An unexpected finding was that administrative agents and administrators had on average greater PTSD symptoms than physicians. Positions providing direct care to COVID-19 patients were not associated with PTSD symptoms. Administrators may have been confronted with multiple decisions that had an impact on the entire structure of care. Second, PCL-5 instructions refer to “response to a very stressful experience”; indeed, symptoms may appear without witnessing actual death. This falls under the aegis of vicarious trauma, which is continuous exposure to others recounting their trauma, reviewing case files or responding to the repercussions of trauma (61). Moreover, a person could have PTSD symptoms that coincide with the survey or have witnessed one of their peers suffer from COVID-19.

Our results are comparable to some similar studies in the literature published since the beginning of the pandemic (11, 57, 62–70). Indeed, among the studies with a similar design as ours, e.g., a cross-sectional study with the aim of verifying factors associated with per-pandemic psychological distress (burnout, psychopathologies) in healthcare workers, the rates of distress and the factors reported are similar to what we present here. Notably, several studies have shown high rates of burnout (64, 66, 67, 69, 70), which are comparable to the 51.8% reported here. However, the factors associated with the development of burnout sometimes differ from the ones we found, with reports of workload, type of employment and participation in training programs, all of which were not found to be significant in our study. However, in this nurse population study, moderate to high levels of burnout were reported and a negative correlation with resilience was found (*r* = 0.25, *p* < 0.05, and *r* = 0.31, *p* < 0.01 for emotional exhaustion and personal inefficacy, respectively) (67). In another study, in primary care physicians, the rate of depression (~14%) was close to the one found here, although factors identified differed as well, with high workload and a single relationship being significant risk factors for depression. However, it has to be mentioned that some other studies showed different results from ours. In particular, two studies (64, 65) showed significantly higher rates of depression and anxiety with prevalence exceeding 45% for depression and 55% for anxiety, a result all the more surprising considering that the scale used was the same as ours (HADS). It should be noted though that the population of these two studies were in a country experiencing civil war (Libya), and the addition of a major stressor such as the COVID-19 pandemic may explain these high rates of psychopathology.

Because self-compassion is a less recognized and studied variable in healthcare compared to resilience, we studied it as part of an exploratory analysis. Our results show that it has a protective effect on anxiety and PTSD, which is consistent with the general population literature (71, 72). Self-compassion refers to the understanding toward one's own feelings and reactions, along with having a well-balanced view when facing difficult situations (73). With the rapidly changing directives regarding daily work during COVID-19 pandemic, difficult emotions and feelings of inadequacy were normative experiences. Self-compassionate participants may have been protected against anxiety and PTSD, with less guilt and self-pressure.

Concerning the limits of our survey study, we cannot establish causal effects, given that we measured our outcome and factors at the same time, making the temporal relationship impossible to assess (74). Also, we may be prone to volunteer bias, as more distressed workers may have been more likely to participate in order to relate their experience; whereas others may not have enough energy to participate. We must take into account another limit in the selection of our participants, as our study was Web-based (75). In fact, this implies that only respondents who were aware of the existence of the survey were able to register, which may lead to under-representation of some specific groups of the population studied. Furthermore, we are aware that other experiences (financial, personal) could have been associated with burnout rate, but chose to focus solely on work experiences. We did so to gather modifiable factors for most hospitals' human resources and because burnout in healthcare workers is mainly driven by organizational and psychological factors with little to no contribution from demographic factors (21, 29, 54–57). Finally, the small sample size of certain groups, such as administrative agents, can limit the external validity. However, the response rate was similar between types of employment and ranged from 70 to 100%, averaging 83 ± 8%. Finally, even if the proportion of women seems high, it is representative of the overall local healthcare workers with 82% being women (76).

Our study differs from previous ones by covering a wide range of variables often treated separately. Notably, this study addresses both organizational and individual psychological health outcomes and considers a combination of factors arising from individual, social and organizational psychology. In addition, our study includes medical workers, but also non-medical health workers facing this pandemic. Our results reinforce the relevance of targeting individuals (77–79) and organizational factors to promote mental health workers facing high-stress situations. Resiliency can be worked on and improved, as it has been previously demonstrated in a meta-analysis on resilience-focused interventions (80). It is important to acknowledge that although one's resilience can be strengthened and worked on, it wouldn't translate into unacceptable environmental conditions being tolerated as a result. For its part, perceived organizational support has been widely researched in the last three decades (32). It encompasses the organization's treatment of its members, employee-organization relationship quality, human resource practices, and job conditions (32). It favors organizational commitment and task performance, general positive affect in the workplace, decreased withdrawal behaviors, turnover intentions, and perception of strain (81). One first step to target this variable in practice would be to analyze whether the source of perceived support is obtained from colleagues, supervisors or, more frequently, from the organization as a whole (82); and then use this channel to promote further interventions. The impact of individual, organizational or both types of intervention would need to be measured prospectively with well-defined targeted health outcomes and populations. Indeed, the present study clearly shows that work environment, employment type, or reassignment are associated with specific mental health symptoms. If future research or clinical initiatives aim toward screening and referring for mental disorders in healthcare workers facing a pandemic, our data suggests that depressive symptoms should specifically be screened for in reassigned workers, whereas PTSD should primarily be screened for in administrative agents, administrators, nurses, other health professionals such as respiratory therapists, beneficiary attendants and laboratory technicians. Ultimately, this research can serve both clinical and research initiatives to support the global psychological health of healthcare workers that are coping with high stress situations. Undeniably, the healthcare system is going through a major crisis with this pandemic being the most important one but probably not the last. Thus, it is likely that the identified factors may have an impact on other healthcare system crises.

## Data Availability Statement

In accordance with the ethical consent provided by participants, the data underlying this article cannot be shared publicly to preserve their privacy.

## Ethics Statement

The studies involving human participants were reviewed and approved by Research Ethics and New Technology Development Committee (CÉRDNT), Montreal Heart Institute, Montréal, Canada. The patients/participants provided their written informed consent to participate in this study.

## Author Contributions

JB, M-FM, J-CT and SG: conceptualization. SC, M-JM, CR, and JB: data curation. CR and M-CG: formal analysis. JB: investigation, project administration, resources, and supervision. SC, M-CG, and JB: methodology. CR and JB: validation. SC, M-JM, and JB: visualization. CR, M-CG, CG, and JB: roles/writing—original draft. All authors recruitment, funding acquisition, and writing—review and editing.

## Conflict of Interest

J-CT reported grants from Amarin, grants and personal fees from Astra Zeneca, grants, personal fees, and other from Dalcor, grants from Esperion, grants from Ionis, grants from Pfizer, grants and personal fees from Sanofi, grants and personal fees from Servier, personal fees from HLS Therapeutics, outside the submitted work; In addition, J-CT had a patent Genetic markers for predicting responsiveness to therapy with hdl-raising or hdl mimicking agent pending, and a patent Methods for using low dose colchicine after myocardial infarction with royalties paid to Invention assigned to the Montreal Heart Institute. The remaining authors declare that the research was conducted in the absence of any commercial or financial relationships that could be construed as a potential conflict of interest.
