# Supplementary material for: Factors Associated With Burnout, Post-traumatic Stress and Anxio-Depressive Symptoms in Healthcare Workers 3 Months Into the COVID-19 Pandemic: An Observational Study
Source: Front Psychiatry. 2021 Jul 8;12:668278. doi: 10.3389/fpsyt.2021.668278 (PMC8295587; doi:10.3389/fpsyt.2021.668278)
Supplement: Supplementary file 1 [file Table_1.docx]

Supplementary Material

# Supplementary Tables

## Appendix 1 — Description of measures (Appendix Table A1).

**Table A1.** Detailed description of the surveyed measures.

| Socio-demographic data | | Age |
| --- | --- | --- |
|  |  | Sex |
|  |  | Gender |
|  |  | Ethnicity |
|  |  | Marital status |
|  |  | Parental status |
| Medical data | | Actual COVID status (+/-/recovered/never investigated) |
| Occupational data | | Health care work type |
|  |  | Work environment |
|  |  | Actual work status |
|  |  | Direct COVID Patient Care |
|  |  | Workload |
|  |  | Number of hours worked per week in the last month |
|  |  | Night shift just before answer questionnaires |
|  |  | Night shift in the last month |
| Organizational data | | Access to adequate personal protection equipment since beginning of COVID pandemic |
|  |  | Perception of security using personal protection equipment |
|  |  | Access to simulation techniques since beginning of COVID pandemic |
|  |  | Last simulation round |
|  |  | Type of simulation |
|  |  | Reassignment |
|  |  | Access to mental health help if needed since beginning of COVID pandemic |
|  |  | Type of professional and or therapy use if needed |
| Psychological questionnaires [self-report questionnaires] | Burnout | Maslach Burnout Inventory [MBI 2; item 8 and 10 of MBI-22: α = 0.80, EE subscale: α = 0.90, DP subscale: α = 0.76, PA subscale: α = 0.82 (Loera, Converso & Viotti, 2014)]  Burnout (yes/no): If the answer to question 8 and/or question 10 is one of the following:  • Once a week  • A few times a week  • Every day  Missing value will be generated in the following situations:  1. If answers to both questions are missing  2. If the answer to one question is missing and to the other question, the answer is less than once per week. |
|  | Resilience | Connor-Davidson Resilience Scale 10-items [CD-RISC-10; α = 0.85 (Campbell‐Sills & Stein, 2007)]  Gross total of all 10 items.  For the score calculation:  Maximum of 3 missing values is accepted.  Missing values will be imputed with the average of the remaining non missing values. |
|  | Anxiety/Depression | Hospital Anxiety and Depression Scale [HADS α = 0.89; HADS-A subscale α = 0.83, HADS-D subscale α = 0.82 (Roberge, Doré, Menear, Chartrand, Ciampi, Duhoux & Fournier, 2013)]  HADS-A: Gross total as defined in the HADS reference document.  For the score calculation, no missing value accepted to the questions related to HADS-A.  Anxiety score will also be categorized as < 11 vs ≥ 11.  HADS-D: Gross total as defined in the HADS reference document.  For the score calculation, no missing value accepted to the questions related to HADS-D.  Depressive symptom score will also be categorized as < 11 vs ≥ 11. |
|  | PTSD | PTSD Checklist for DSM-5 [PCL-5; α = 0.94 (Ashbaugh, Houle-Johnson, Herbert, El-Hage, & Brunet, 2016)]  Adjusted PTSD score = Gross total score x 20 / (total number of items answered [≥ 18]).  Maximum of 2 missing values is accepted for the calculation of PTSD score.  Adjusted PTSD score will also be categorized as < 31 vs ≥ 31. |
|  | Self-compassion | Self-compassion Scale [Neff; α = .91 (Leary, Tate, Adams, Batts Allen, & Hancock, 2007)]  Gross total of 3 items (*I try to be understanding and patient towards those aspects of my personality I don’t like; When I feel inadequate in some way, I try to remind myself that feelings of inadequacy are shared by most people; When something painful happens I try to take a balanced view of the situation*).  No missing value accepted. |
|  | Social support | Social Support Questionnaire - Satisfaction [SSQ; α = 0.79 (Sarason, Sarason, Shearin, & Pierce, 1987)]  SSQ-Satisfaction: Gross total of questions 1b, 2b, 3b, 4b, 5b, 6b.  For the score calculation:  Only 1 missing value is accepted.  Missing value will be imputed with the average of the remaining non missing values. |
|  | Perceived Organizational Support | Perceived Organizational Support Scale [POS; α = 0.93 (Worley, 2006)]  Gross total of all 8 items.  For the score calculation:  Maximum of 2 missing values is accepted.  Missing values will be imputed with the average of the remaining non missing values. |
